# Advanced deep learning techniques for recognition of dental implants

**DOI:** 10.1016/j.jobcr.2025.01.016

**Published:** 2025-02-08

**Authors:** Veena Benakatti, Ramesh P. Nayakar, Mallikarjun Anandhalli, Rohit sukhasare

**Affiliations:** a Dept of Prosthodontics and Crown and Bridge, KAHER’S KLE VK Institute of Dental Sciences, Belagavi, Karnataka, India; b Department of Electronics and Communication Engineering, Central University of Karnataka, Kalaburagi, Karnataka, India; c Project research scientist, KAHER’S KLE VK Institute of Dental Sciences, Belagavi, Karnataka, India

**Keywords:** Dental implants, Deep learning, DEtection TRansformer, Radiographs

## Abstract

**Background:**

Dental implants are the most accepted prosthetic alternative for missing teeth. With growing demands, several manufacturers have entered the market and produce a variety of implant brands creating a challenge for clinicians to identify the implant when the necessity arises. Currently, radiographs are the only tools for implant identification which is inherently a complex process, hence the need for implant identification technique. Artificial intelligence capable of analysing images in a radiograph and predicting implant type is an efficient tool. The study evaluated an advanced deep learning technique, DEtection TRanformer for implant identification.

**Methods:**

A transformer-based deep learning technique, DEtection TRanformer was trained to identify implants in radiographs. A dataset of 1138 images consisting of five implant types captured from periapical and panoramic radiographs was chosen for the study. After augmentation, a dataset of 1744 images was secured and then split into training, validation and test datasets for the model. The model was trained and evaluated for its performance.

**Results:**

The model achieved an overall precision of 0.83 and a recall score of 0.89. The model achieved an F1-score of 0.82 indicating a strong balance between recall and precision. The Precision-Recall Curve, with an AUC of 0.96, showed that the model performed well across various thresholds. The training and validation graphs showed a consistent decrease in the loss functions across classes.

**Conclusion:**

The model showed high performance on the training data, though it faced challenges with unseen validation data. High precision, recall and F1 score indicate the model's potential for implant identification. Optimizing this model for a balance between accuracy and efficiency will be necessary for real-time medical imaging applications.

## Graphical abstract

**Figure:**
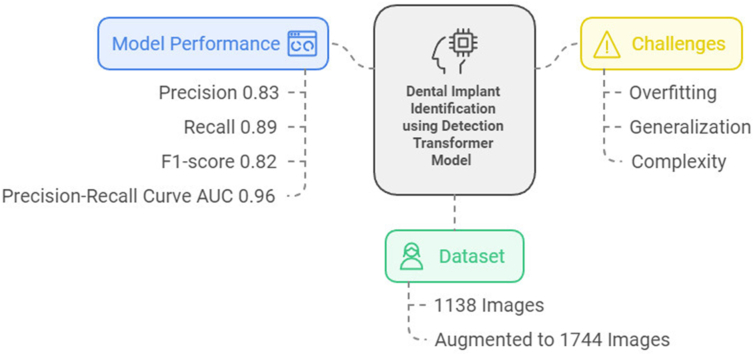

## 1 Introduction

Oral health contributes to the general and social well-being of individuals. In particular, the missing teeth can affect the quality of life and individual societal roles.^1^ Several attempts have been made in history to replace missing teeth, the modern implantology has shown promising alternatives for the same. Branemark laid down the concept of direct bone-implant contact through osseointegration using titanium. With the benefits offered, currently, dental implants are the most advanced and accepted form of replacement alternative for missing teeth.^2^

Demand for dental implants is increasing leap by leap globally and is expected to grow manifold in the coming years.^2^ Dental implant usage increased four-fold from 1983 to 1987 in the United States, further by 75 % between 1986 and 1990. At the beginning of this century, 25 dental implant manufacturers were marketing around 100 implant systems with varied lengths, diameters, surfaces, interfaces, platforms, and body shapes. The distinctions were made on the implant abutment interface, shape, and implant-to-bone surface.^3^ Continued demand for dental implants has led many implant manufacturers to enter the industry and produce more than 300 implant brands and the variety continues to evolve. Each of these companies produces many variants that vary in lengths, diameters, surfaces, platforms, interfaces, body shapes, morphology, connections, and surface characteristics.^3^ This wide variety and unique design make it challenging for clinicians to identify the implant system when the necessity arises. With changing demands and trends, newer varieties are continually introduced, and the older ones become obsolete, adding to the complexity of implant identification when dealing with obsolete models.

Dental implants need aftercare and follow-ups due to biological and mechanical complications and these complications may arise any time after placement even several years later.^4^ During this information about the implant brand is required. Implant systems are designed uniquely for the company and need specific tools or components. The implant identification process becomes challenging when records are unavailable and patients seek treatment from different clinicians in different regions or countries. With the growing trend of dental tourism, clinicians sometimes have to manage implants placed elsewhere which could be of an exotic system that cannot be recognized easily. These situations challenge even experienced clinicians which may lead to ethical complications.^4^ Presently, patient records and radiographs are the only tools for identifying implant systems that require significant time and effort, yet remain an assumptive process.

The implant identification process also plays a significant role in revealing the person's identity in forensic dentistry. Several approaches have been proposed in the literature however none of them are easy or quick and consume a significant amount of human effort, knowledge, experience, and time. With the continued thrust for implant identification researchers have turned towards artificial intelligence (AI) to identify implants in an image. Deep learning (DL) a subset of AI is efficient in object detection and classification, this can be adopted to identify implants in a radiograph.

## 2 Methodology

The study got approval from the Institutional Review Board (KAHER/EC/21–22/D-290721002). As the study was non-interventional in design and collected anonymized data, an ethical waiver was obtained by the Ethical Board.

### 2.1 Proposed model

Several deep learning models have been explored for implant identification, but DEtection TRansformer (DETR), a transformer-based DL model remains unexplored. DETR architecture changes the game in object detection with a transformer-based framework that replaces anchor boxes and non-maximum suppression.^5^ It takes the CNN backbone, such as ResNet-50, to extract feature maps that are then processed by a transformer encoder that captures global relationships through multi-head self-attention.^6^ DETR uses bipartite matching with the Hungarian algorithm along with a set-based loss that combines classification, L1 regression, and generalized IoU, thereby making the predictions per image non-redundant of fixed numbers.^7^ This design has opened for global reasoning over an image resulting in better performances for complex detection tasks. Extensively tested on COCO and applied to panoptic segmentation, it shows effectiveness and versatility.^8^

### 2.2 Training of DETR model

Once the data was secured, data cleaning was executed which is crucial to the training process that helps ensure accuracy and consistency by labelling, removal of irrelevant data and duplicates, reduction of noise and enhancement of images through OpenCV techniques. Availability of certain implant classes was limited; hence we considered strategies like adjusting class weights, fine-tuning, and choosing appropriate loss functions to achieve effective training.

Following data cleaning accurate and consistent labels (type of implant system) were assigned using makesense.ai. Then the dataset was divided into training (70 %), validation (15 %), and test datasets (15 %). The training set was used to make the model by learning and the validation set to assess the model during the training process while the test set evaluated the final trained model.

The model was trained on Google Colab for 50 epochs with 16 GB NVIDIA Tesla T4 GPU, focusing on detection over five classes. It then took its first round of training without any augmentations. On evaluation, we found that the model exhibited confusion between the classes and the performance was not optimal. The second run included data augmentation, which consisted of rotation, flipping, scaling, cropping, and colour adjustments. This increased the dataset to 1744 images and then the model was retrained and evaluated for performance.

## 3 Results

### 3.1 Dataset

A total dataset of 1300 radiographs which included 950 intraoral periapical radiographs (IOPA) and 350 orthopantomograms (OPG) was initially collected from clinicians and institutions. Radiographs with unknown implants, distortion, blur, and other conditions that hinder the clinical recognition and classification of dental implant systems were excluded. After eliminating unusable data, the dataset comprised 1138 radiographs, including 868 IOPA and 270 OPG. Different implant brands included were, Osstem TSIII SA, Dentium superline, Noris Tuff TT, MIS V3 and Adin Touareg. DETR was trained on these subvariants of implant systems focusing on identifying these specific types.

### 3.2 Performance metrics

The DETR's performance was evaluated using metrics, precision, recall, an ROC curve, and detailed training and validation graphs.

**Fig. 1:**
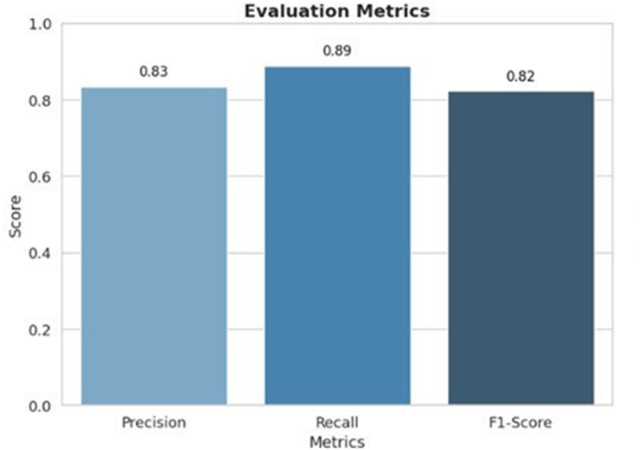
Evaluation results for the DETR.

Fig. 1., demonstrates the evaluation results of the DETR model in identifying five implant types. Key performance metrics such as precision, recall, and F1-score are presented for an overview of model efficacy in multi-class classification.

For the DETR model, excellent detection performance was exhibited for five classes of dental implants. The model achieved an average precision of 0.83 and a recall of 0.89, which implies that 89 % of true implant types were detected accurately. The model achieved an F1-score of 0.82 indicating a strong balance between recall (detecting implants) and precision (minimizing false positives), making the model well-suited for applications like dental implant detection. The F1 score gives a comprehensive performance of the model in detecting implant systems. These results demonstrate the DETR model's ability to accurately detect dental implants while maintaining a balance between precision and recall.

Analysis of training and validation processes, including cardinality errors and loss functions, provided insights into model behaviour. These results validate DETR's potential for accurate, multi-class dental implant detection.

**Fig. 2:**
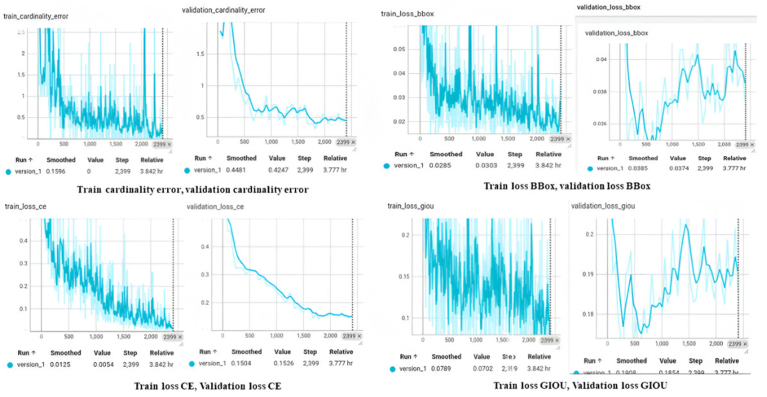
Comparative analysis of training and validation - cardinality error, loss BBox, loss CE, loss GIOU.

Cardinality error measures the difference between the number of predicted implants and the actual (ground truth) implants. It is critical for object detection models like DETR which predict multiple objects at once. Train cardinality error measures this error during the training phase while the validation cardinality error measures this error during the validation phase. The graph illustrating training and validation cardinality errors (Fig. 2) indicates that the training error decreases to 0.1596 by step 2399, whereas the validation error reduces to 0.4481. The smooth validation curve suggests effective generalization, indicating improved predictions of implant counts over time.

From Fig. 2., Train loss BBox (left) and validation loss BBox (right) show bounding box prediction. This loss represents how well the model predicts the coordinates of the bounding boxes around implants. Training loss lowers down to 0.0285 (smoothed) and 0.0303 (unsmoothed) as it shows improved performance through the fluctuations that occurred earlier. Validation loss was reduced to 0.0385 (smoothed) and 0.0374 (unsmoothed) with consistent improvement and good bound-box prediction. The smoothed value depicts the average error of all the steps showing the overall trend while the unsmoothed shows the error at the final step.

Cross-entropy (CE) loss is the measure of how well the model can classify implants within bounding boxes. From Fig. 2, the training CE loss decreased to 0.0125 which means better classification. Validation CE loss decreased to 0.1504 (smoothed) and 0.1526 (unsmoothed) and was higher than training loss but without overfitting. The closely aligned curves reflect the strong model performance and generalization for implant classification.

While IoU (intersection over union) measures the overlap of the ground truth and predicted boxes, GIoU provides a better optimization signal by penalizing non-overlapping boxes more effectively. Here the goal is to minimize GIoU loss to get tighter, better-aligned bounding boxes. Training Loss GIoU (left) and Validation Loss GIoU (right) from Fig. 2., show specific loss functions used in bounding box regression. Training loss begins from fluctuations and goes down to 0.0789 smoothed (average values over time, reducing noise) and 0.0702 unsmoothed (raw values, showing exact fluctuations) at step 2399 which means that the predictions for bounding boxes are being improved. However, from step 1500 the validation loss goes up 0.1864 smoothed and 0.1952 unsmoothed, implying reduced generalization to new, unseen data for overlapping boxes.

**Fig. 3:**
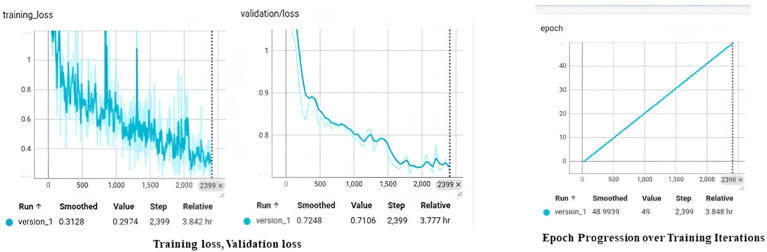
Comparative analysis of Training and validation - Loss and Epoch Progression.

The first graph from Fig. 3., shows Training Loss and Validation Loss. Both graphs plot loss values (y-axis) against the number of iterations in the learning process (x-axis). This represents the overall loss for the training and validation phases. Training Loss shows a continuous decrease from ∼1.2 to 0.2974. The validation loss appears smoother, with a progression from ∼1.0 to 0.7106. Both plots continue to demonstrate a positive and consistent direction, thus describing the model's positive learning progression.

In Fig. 3., the second graph shows epoch progression. The trend line for "version_1″ has a uniform positive slope, ending at (2,399), with a smoothed epoch value of 48.9939, rounded to 49. This is a linear trend that reflects constant, uniform growth in learning or data without stagnation and reversal.

**Fig. 4:**
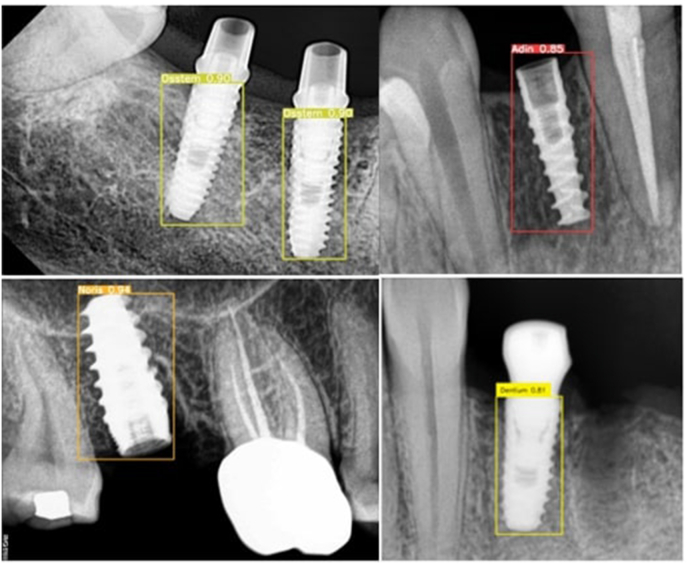
Test images with prediction.

For optimal model performance, minimization of all losses is necessary to ensure accurate bounding box predictions and object classifications. By tracking these metrics, we can identify where the model is improving or where it might need more fine-tuning or adjustments. Fig. 4, illustrates the test images where DETR identifies the implant systems with confidence levels.

## 4 Discussion

In the evolving field of implant dentistry, the identification of implant brands is a critical step for successful implant maintenance. Until now there is no. one well-established method for this purpose, researchers have turned towards artificial intelligence leveraging its object recognition capability through deep learning techniques. Many state-of-the-art Deep learning models are utilized for this purpose.^9^, ^10^, ^11^, ^12^, ^13^ The DETR model used in our research holds immense significance as it exhibits unique architecture suited for object recognition.

The deep learning models analyse an image in the radiograph to automatically extract features and assign respective classes. In the context of implant identification, DETR distinguishes implant types on their unique features such as shape, design, thread, pitch, apex geometry, collar, and overall implant geometry. These features extracted from radiographic images serve as learning patterns for the model.

Transformer models have emerged as a revolutionary concept in AI for object detection with exceptional performance. DETR is a prediction model that uses a transformer encoder-decoder architecture to predict all objects at once. DETR combines a CNN backbone for feature extraction, a transformer encoder-decoder for refining features, and a feed-forward network (FFN) for detection predictions. DETR comprehensively processes the whole image allowing the model to predict bounding boxes and classes simultaneously. This approach enhances the model's ability to interpret complex images effectively.^14^

Ickler et al. investigated the feasibility of DETR models, such as conditional DETR and DINO DETR, that have been applied to a variety of volumetric medical object detection tasks on different datasets which include anomalous regions in anatomical organs, and lung nodule detections. The fact that DETR models surpass other traditional anchor-based methods, such as Retina U-Net, on most datasets suggests that they have great potential. Since DETR models eliminate manual heuristics such as anchor designs and non-maximum suppression, they simplify and strengthen the detection process. DETR, despite their potential, is less studied, hence we considered assessing its ability in implant identification.^15^

### 4.1 Performance metrics

The DETR model has proven to be strong in detecting and classifying dental implants into five categories in the validation dataset. The overall precision of 0.83, recall of 0.89, and F1-score of 0.82 indicate a good balance between false positives and false negatives, making this model a good competitor for clinical deployment in dental imaging applications. These encouraging results suggest that the DETR model could detect and classify dental implants with high accuracy while also generalising over different types of dental implants. Apart from this performance metric, there lies an imperative sense to analyse some of the aspects behind the training process and validation for the model. This analysis can give insight into its behaviour, particularly cardinality errors, loss functions, and other related factors that might affect its detection performance.

### 4.2 Error study

The cardinality error results show that although the training error has dropped to 0.1596, which is a good object count prediction, the validation error remains higher at 0.4247, which suggests overfitting. This shows that the model has a hard time generalizing to unseen data, which is an important requirement in medical applications where accurate predictions are important. Although the model shows promise, further work is needed to reduce the gap between training and validation performance. Generalization will be very important for the model to predict well across a variety of patients, imaging conditions, and types of implants in clinical settings.

The analysis demonstrates that the training loss consistently decreases to 0.2974, while the validation loss is high at 0.7106, which means that it performs better on training data than on unseen data, suggesting overfitting, a very common problem in complex applications such as medical imaging. The validation curve emphasizes the need for techniques to improve the generalization of the model beyond the training set.

The model requires a training time of 3.8 h, and its complexity may be due to data volume or architecture depth. Despite considerable improvement in training loss and error metrics, validation errors remain high, thus needing better generalization. Methods such as regularization, cross-validation, hyperparameter tuning, and more diverse training data will prevent overfitting and improve the robustness of the model for actual clinical use.

While the model has good performances on the training set, overfitting and generalization to unseen data would be an issue, and model complexity will also probably lead to a higher computational time and more memory usage. Optimizing this model for some balance between accuracy and efficiency will be necessary for such real-time medical imaging applications.

### 4.3 Comparative analysis

When compared to previous research in the field of dental implant identification, the DETR model also showed promising results maintaining high performance comparable to CNN models, but there are key differences in approach and capabilities. Many previous studies have relied on traditional CNN-based architectures,^9^, ^10^, ^11^, ^12^,^9^, ^10^, ^11^, ^12^ such as YOLO,^13^^,^^16^ Faster R-CNN^17^ or RetinaNet, which excels at object detection tasks but often struggles with the spatial relationship between objects and context within the image.

CNNs can efficiently be used in real-time applications and small datasets due to their power in feature extraction, capturing local patterns, and spatial hierarchies. CNNs perform well in tasks that use critical local features such as image segmentation and anomaly detection. The U-Net and Retina U-Net models are applied for medical image segmentation, as they are computationally efficient for processing smaller data sets.^18^^,^^19^ However, the CNNs do not model long-range dependencies; therefore, they fail to process complex, large-scale images.^19^

Transformer models utilize self-attention mechanisms, which give deep contextual understanding with the realization of global contexts and very long-range dependencies, showing advantages in tasks involving deep spatial understanding. This thus gives transformers an edge in cases involving complex object detection and high-resolution image classification needs, which CNNs fail to address. The anchor boxes or manual heuristics in the object detection pipeline are removed by DETR models, and they outperform CNN-based detectors like Retina U-Net in various benchmarks, such as RibFrac, KiTS19, and LIDC.^18^^,^^20^ However, transformers need significantly larger datasets and more computing power than CNNs to fully exploit the advantages of transformers.^18^

Further, transformers work better in global context scenarios and have shown robustness in the context of continual learning where they are able to learn new data without forgetting the previously learned information.^18^ This makes them a good fit for dynamic environments where medical data changes over time, which CNNs cannot handle because they rely on static patterns.^19^ Hybrid models like ConvFormer, which combine the best of both architectures, balance local feature extraction within CNNs with global context modelling in transformers, thus achieving state-of-the-art results in medical imaging tasks.^19^^,^^20^

In the context of implant recognition, DETR exhibits certain advantages, DETR's transformer-based mechanism captures spatial relationships and contextual information between different implant types whereas conventional models rely only on CNN layers failing to understand complex interactions. DETR processes the entire image at once focusing on the global context with finer details whereas other models have a fixed approach relying on localised features struggling to retain a global context. The disadvantages of DETR over traditional models include model complexity leading to a higher computational time making it slower and more memory usage, and the need for larger and more diverse datasets for better performance.^19^^,^^20^

### 4.4 Limitations of study

The results are limited to the datasets that were available for the study. We considered only five implant systems considering the data availability issues. Working with a broad range of implant systems and larger and more diverse datasets would help in developing a robust model.

### 4.5 Future recommendations

Future work should focus on improvising the model's generalization capabilities by implementing cross-validation, regularization techniques, and hyperparameter tuning, along with larger and more diverse training datasets.

## 5 Conclusion

The DETR model outperforms in dental implant detection and classification but there is a scope for improvement in generalization, given by validation loss and cardinality error. Optimization with the model using regularization, cross-validation, hyperparameter tuning, and data augmentation could be done in future work. Increasing the dataset size and fine-tuning the model for complex variation can further help in real-world usability. The model would be developed further and thus can become a useful tool for automating dental implant detection, improving diagnostic accuracy, and reducing clinician workload.

## Patient's/Guardian's consent

The data used in this research was fully anonymized before access by our research team. No personally identifiable information was collected, stored, or processed during the course of the study. As no direct patient interaction or intervention was involved, a waiver of consent was obtained by the institutional review board. The study complies with all institutional ethical guidelines and data protection standards.

## Ethical

As the study was noninterventional in design and collected anonymized data, an ethical waiver was obtained by the Ethical Board, (KAHER/EC/21–22/D-290721002).

## Data availability

The dataset used in this study is not publicly available. However, it can be made available upon request, subject to verification.

## Funding disclosure

This research was supported by financial grants from the Indian Council of Medical Research (ICMR), New Delhi, India, for the duration of three years with file bearing no 5/4/2–16/OH/2023-NCD-II. The study is part of a funded project under these grants, aimed at advancing the application of artificial intelligence in dental implant identification.
